# Health literacy and depression in women with type 2 diabetes mellitus

**DOI:** 10.6061/clinics/2020/e1436

**Published:** 2020-05-18

**Authors:** Yu-Ling Hsu, Deng-Huang Su, Su-Chen Kuo

**Affiliations:** I Department of Health, Taipei City Government, Taipei, Taiwan.; II Department of Internal Medicine, Far-Eastern Polyclinic, Taipei, Taiwan.; III College of Nursing, National Taipei University of Nursing and Health Sciences, Taipei, Taiwan.

**Keywords:** Health Literacy, Depression, Self-Rated Health Status, Diabetes Mellitus

## Abstract

**OBJECTIVES:**

The prevalence of diabetes mellitus has recently increased in Taiwan, and depression is common among these patients. Moreover, a lack of health literacy may lead to depression. In this study, we explored the correlation between health literacy and depression in diabetic women.

**METHODS:**

In this cross-sectional study, 152 women with type 2 diabetes mellitus were recruited from the outpatient clinic of a regional teaching hospital in Taiwan. The data were collected through medical records and a self-reported structured questionnaire, which included items on basic attributes, self-rated health status, the Center for Epidemiologic Studies Depression Scale (CES-D), and Chinese Health Literacy Scale for Diabetes (CHLSD). The results were analyzed using descriptive statistical analyses, bivariate correlation tests, and linear regression analyses.

**RESULTS:**

One hundred thirty-five valid questionnaires were obtained. Approximately 20% of the participants had a higher tendency toward depression as per their CES-D score, and the CHLSD results showed that 13.33% had poor health literacy. There was a negative correlation between health literacy and depressive tendencies after adjusting for self-rated health status, economic satisfaction status, employment status, and education level using multivariate linear regression analyses. For each 1-point rise in the CHLSD score, the CES-D score decreased by 0.17 points (*z*=−2.05, *p*=0.042).

**CONCLUSIONS:**

A negative correlation was identified between health literacy and depression. Self-rated health status, economic satisfaction, employment status, and higher education level are factors that also affect depressive tendency among diabetic women.

## INTRODUCTION

In recent years, there has been a significant upward trend in the prevalence of diabetes mellitus (DM) in Taiwan. The number of women with DM has increased from 3.34% of the total population in 2000 to 5.24% in 2009. In 2009, the prevalence of DM in 40-59, 60-79, and ≥80-year-old women was 5.47%, 21.97%, and 23.97%, respectively (1). DM is a chronic disease with complications involving multiple organs; 21.3% of patients develop retinopathy; 6.2%, cardiovascular disease; 2.3%, cerebrovascular disease; 0.6%, complications requiring amputation; and 0.67%, blindness. Unfortunately, 1.55% of patients eventually suffer from end-stage renal disease (2).

Depression is common among diabetic patients, with a prevalence of 50% in certain countries (3). A higher medical cost and tendency towards suicide, hospitalization, and poorer life quality was found in diabetic patients with depression than in those without depression (4-6). Diabetic women may be prone to depression (7). Men with DM and depression had higher in-hospital mortality than did women, especially for older patients and patients with multiple diseases (8). Taken together, these results indicate that depression is a common comorbidity and can have adverse consequences among diabetic patients.

Health literacy (HL) is the ability to acquire, read, understand, and apply knowledge of health care. People who are health literate can make appropriate health choices and follow treatment recommendations (9). Thus, HL includes both knowledge and action. Poor HL may lead to insufficient health-related knowledge, poor disease self-management, and non-use of health services, as well as further affecting patient health and survival status. In diabetic patients, poor HL is associated with a lack of health knowledge of DM and may be associated with healthy outcomes (10). A study of functional HL of patients with type 2 DM in Brazil showed that one-fourth of the participants had problems with their HL skills; 11.3% had borderline deficiency in their skillset, and 15.3% had insufficient skills (11). Especially for diabetic patients who were taking antidepressants, the percentage of low HL was as high as 72% (12). Fortunately, HL among diabetic patients can be improved through instruction (13,14) and improving their communication with others (15) or knowledge of DM (16).

One research question of interest is whether the level of diabetic HL is related to depression among diabetic patients. Improving mental HL temporarily reduced the severity of depression symptoms (17). Low education level and HL are related to depression and declining cognitive ability (18). Sex, marital status, and income affect a person’s degree of depression and HL (19). However, few studies have been conducted on the correlation between HL and depression among diabetic women. This study explored the correlation between HL and depression of diabetic women.

## MATERIALS AND METHODS

### Study patients and design

This was a cross-sectional study of diabetic women who were recruited from the outpatient clinic of a regional teaching hospital in north Taiwan. The inclusion criteria were as follows: (1) adult women aged 20 or older with type 2 DM; (2) awareness, cognition, visual and hearing function, and oral expression at levels that do not prevent communication; and (3) ability to read. The diagnosis of type 2 DM was based on a physician’s’ diagnosis and patients’ medical records. This study excluded patients with type 1 DM, pregnant women, aboriginal patients, and patients on dialysis. In Taiwan, a diagnosis of type 1 DM is recorded in their health ID card, so the patients with type 1 DM could be confirmed by their medical records. The study was approved by the Institutional Review Board of the Landseed Hospital, Taiwan (approval number: 16-017-B1).

In this study, 152 women were recruited between July 1 and August 31, 2016. Their written, informed consent was obtained. Data were collected using medical records and a self-reported, structured questionnaire, which included items on basic attributes and self-rated health status in addition to the Center for Epidemiologic Studies Depression Scale (CES-D) and Chinese Health Literacy Scale for Diabetes (CHLSD). The dependent variable was the degree of depression of these patients, which was measured using the Chinese version of the CES-D (20). The participants recalled their subjective feelings and behavior over the previous week. There were 20 items, and each was scored on a 4-point Likert scale, totaling 60 points. A score of more than 16 points was associated with a depressive tendency. The Cronbach's alpha for the general population and clinical patients was 0.85 and 0.90, respectively (21). Regarding the questionnaire’s validity, the mean score (24.42) of the patients with mental illness was higher than that (7.94-9.25) of the general population. Moreover, a CES-D score of more than 16 in patients with mental illness (70%) was significantly more common than in the general population (15%-19%) (21).

The main independent variable was the level of HL, which was measured using the multiple-choice version of the CHLSD (22,23). The CHLSD comprises four components (remember, understand, apply, and analysis) with 34 items. The highest score is 68 points, and less than 52 points is considered insufficient diabetic HL (23). The Cronbach's alpha for the overall scale and the scale’s individual components was 0.884, 0.885, 0.667, 0.654, and 0.717, respectively. Regarding the validity of the CHLSD, the correlations between the CHLSD score and the scores of the Diabetic Knowledge Scale, Diabetic Management Self-Efficacy Scale, Preschool and Primary Chinese Literacy Scale, and Chinese Value of Learning Scale were 0.398, 0.257, 0.822, and 0.303, respectively (23). The scale was originally published in Hong Kong Chinese, and some minor modifications were made for our study after Professor Leung’s agreement.

Participant age was calculated as 2016 minus the year of the participant’s birth. Education level was divided into elementary school, junior high school, senior high school, and college or above. Marital status was categorized as unmarried, married, widowed, and divorced. Status of religious belief was divided into yes or no. Employment status was divided into unemployed, employed, and housewife. Monthly income was divided into three levels: no income, less than 38800 New Taiwan Dollars (NTD, USD 1208, exchange rate USD=NTD 32.13), and higher than 38800 NTD (USD 1208). Economic satisfaction was categorized into four levels: living beyond income, living at income level, living within income, and living well within income. The term “living beyond income” is used to depict the subject’s opinion, that the money that one spends is more than their income. Similarly, other terms are used for the subject’s feeling “money that one spends is just as his income,” “he has some after his money spent,” and “his income is far more than his money spent,” respectively. Dependence status was divided into two levels: dependent on others, and having self-care abilities. The companions of the participants were either family members or other people.

Duration of DM and usage of insulin were based on the patients’ medical reports. Favorable sugar control was based on a level of glycated hemoglobin (HbA1c) of less than 7%. HbA1c was used primarily to identify the 3-month average plasma glucose concentration. The patients self-rated their health status using a visual analog scale, with 0 indicating very bad and 10 indicating very good.

### Statistical analysis

The results were analyzed using descriptive statistical analyses, bivariate correlation tests, and linear regression analyses. For bivariate correlation tests, if the bivariate variates were continuous, the Pearson’s correlation coefficients were verified. When one variable was a binary variable and the other was a continuous variable, an independent t-test was used to test their correlation if the continuous variable was normally distributed; otherwise; the Kruskal–Wallis test was employed. When the categorical variable had more than two levels, the Kruskal–Wallis test was used.

Uni- and multivariable linear regression models were finally used to test the correlation between depressive tendency of the participants and their HL. Model selection with stepwise procedures was used to construct the final model with confounding factors. The criteria for model selection was *p*<0.15. We set *p*<0.05 as statistically significant. All the statistical analyses were performed using SAS 9.2 (SAS Institute, Cary, NC, USA).

This study explored the correlation between the depressive tendency of diabetic women and their HL, which was influenced by many other confounding factors. Therefore, it was possible to estimate the size of samples using a rule of thumb. This rule is that 10 events can determine a variable. The estimated prevalence of depression in diabetic women is 30%, and three confounding factors were included in the linear regression models in addition to HL. The estimated sample size was approximately 134. Additionally, 10% of cases were expected to be excluded and, therefore, at least 148 participants would be recruited in this study.

## RESULTS

### Demographic and clinical characteristics of the patients

In this study, questionnaires were issued to 152 women; 8 questionnaires were returned incomplete, no blood samples were obtained for 8 women, and one woman was discovered to have type 1 DM, all of which led to exclusion. Thus, 135 valid questionnaires were obtained.

The demographic characteristics of these patients are summarized in Table 1. Their age range was 22-87 years (average, 54.66±11.43 years). There were 19 (14.07%) participants whose age was more than 64 years. The age of most participants’ (56, 41.48%) was between 55-65 years. Only 22 (16.30%) participants were younger than 45 years. Regarding their education level, 29 (21.48%), 28 (20.74%), 44 (32.59%), and 34 (25.19%) had elementary school, junior high school, senior high school, and college or above education levels, respectively. A total of 66 (48.89%) were professionals, 47 (34.81%) were housewives, and 22 (16.30%) were unemployed. Only 18 (13.33%) of the women had monthly income higher than USD 1208, and 40 (29.63%) had a monthly income of less than USD 1208. Two persons (1.48%) described themselves as living well within their income, 34 (25.19%) as living with their income, 86 (63.70%) as living at their income level, and 13 (9.63%) as living beyond their income.

The participants’ known duration of DM was 1-32 years (mean, 7.2±6.12 years). The duration of DM of 104 women (77.04%) was less than 10 years. Oral antidiabetic drugs were their main treatment drugs, and only 33 women (24.44%) had been prescribed insulin. Their HbA1c was 5.2%-14.7% (mean, 7.91%±1.69%). Only 42 women (31.11%) had HbA1c≤7%. The range of CES-D scores was 0-36 points (mean, 10.87±8.4 points). There were 28 patients (20.74%) whose CES-D score was not fewer than 16 points. The range of CHLSD scores was 14-68 points (mean, 59.7±8.55 points). The CHLSD scores of 18 patients (13.33%) were not more than 53 points. The range of self-rated health status was 0-10 points (mean, 5.89±1.70 points).

In summary, the patients were mid-aged, mostly high-school-educated, and married, with religious beliefs. Approximately 40% had jobs, and 35% were housewives. Most of the women were relatively satisfied with their economic situation, lived with family members, and were self-dependent. Most of the patients have had DM for less than 10 years. They were usually prescribed oral hypoglycemic agents, and HbA1c level was >7% in approximately 70% of cases. The average self-rated health score was 5.89 points. Approximately 20% of the women had a depressive tendency, and 13.33% had poor diabetic HL.

### Correlation between CES-D score and other variables

Whether CHLSD score, self-rated health status, economic satisfaction, marital status, and employment status were correlated with depressive tendencies in diabetic women was assessed using the criterion *p*<0.15. The results are listed in Tables 1 and 2.

The Pearson’s correlation coefficient between CES-D and CHLSD score was −0.181 (*p*=0.036), indicating that higher CHLSD scores were weakly correlated with lower CES-D scores. The Pearson’s correlation coefficient between CES-D score and self-rated health status was −0.280 (*p*=0.001), indicating that if a participant felt healthy, she would have a lower depressive tendency. Regarding economic satisfaction, the mean CES-D score of the combined living beyond income + living well within income group was 18.13 points, much higher than the mean CES-D score (9.96 points) of the combined living at income level + living within income group. The test value of the Kruskal–Wallis test was 7.533 (*p*=0.007) and followed a chi-squared distribution with one degree of freedom. However, the CES-D score was not correlated with income. This result showed that depression was related to psychological satisfaction, not to true income. For marital status, the mean CES-D score of the combined unmarried and widowed group was 14.15 points, which was not significantly higher than the mean CES-D score (10.30 points) of the combined married and divorced group. The test value of the Kruskal–Wallis test was 2.501 (*p*=0.11) and followed a chi-squared distribution with one degree of freedom. The mean CES-D score of the unemployed women was 13.95 points, whereas that of the combined employed women and housewives group was 10.27 points. The test value of the Kruskal–Wallis test was 3.464 (*p*=0.063). No significant correlation was discovered between CES-D score and other variables.

### Linear regression analyses of depressive tendency in women with diabetes

HL was negatively related to depressive tendency in diabetic women, even after adjustment for confounding factors. Self-rated health status was also negatively related to depressive tendency. College education or higher, unemployment, and living beyond income or living well with income were positively related to the depressive tendency in this study. The results of linear regression analyses are listed in Table 3.

In univariate analyses, only HL, self-rated health status, and economic satisfaction were significantly correlated with the CES-D score. Marital and employment statuses had a near-significant relation with the CES-D score, but other factors had no relation. In multivariate analyses, the final confounding factors in our models were self-rated health status, economic satisfaction, employment status, and education level. The CHLSD score had a negative correlation with the CES-D score, and the results showed that an elevation of one-point in the CHLSD score was related to a decrease of 0.17 points in the CES-D score (*z*=−2.05, *p*=0.042); this indicates that increasing HL of these patients may lessen their depressive tendency.

Economic satisfaction was highly correlated with depressive tendency in diabetic women, and the adjusted CES-D score of the combined living beyond income + living well within income groups was 8.27 points higher than that of the combined living at income level + living within income groups (*z*=3.92, *p*=0.0001). This showed that people who are satisfied with their economic condition tend to have a lower depressive tendency. Similarly, self-rated health status was negatively related to the depressive tendency, and the adjusted CES-D score would be decreased by 1.11 points with a 1-point elevation in self-rated health status (*z*=−2.85, *p*=0.005). Thus, the participants were happier if they felt that they were healthy. Furthermore, the adjusted CES-D score was 3.74 points lower among diabetic women who were unemployed compared with the professional women and housewives (*z*=−2.03, *p*=0.044). Education level was related to HL, so this variable may be a confounder in the relationship between HL and depressive tendency. In our study, a higher education level (college or above) was related to a higher depressive tendency. The adjusted CES-D score of the participants with an education of college level or above was 2.88 points higher than that of those with less education (*z*=1.83, *p*=0.069).

## DISCUSSION

HL and depressive tendency were negatively correlated in diabetic women in this study. Self-rated health status, economic satisfaction, employment status, and education level were also related to depressive tendency.

A study of factors of depression in Chiayi, Taiwan, revealed that 20% of diabetic women and 15% of diabetic men had depression (24), whereas a study in Spain discovered found a prevalence of 15.1% and 5.2%, respectively (4). In a study of hospitalized patients in Spain, the percentage of diabetic men with depression increased from 3.54% in 2001 to 5.22% in 2011, and the equivalent percentage of women rose from 5.22% in 2001 to 9.24% in 2011 (8). The majority of studies have demonstrated that women with DM have a higher prevalence of depression than men (24-28). The factors of depression may be different for women and men; therefore, here we only focused on diabetic women.

Inadequate HL was related to depressive tendency. An Australian study involving 224 diabetic patients discovered that lower education levels, being an immigrant, and depressive emotions were associated with lower HL (29). A study of the general population in South Korea demonstrated that people with lower HL had significantly more depressive symptoms (30). Another study involving 1724 patients sampled from the Healthy Aging in Neighborhoods of Diversity Across the Life Span Study in the United States revealed that age, HL of less than grade V, and education level below senior high school were significantly related with depressive symptoms (18). A study of 3260 elder persons showed that the adjusted odds ratio of depression of persons with a lower HL is 1.2 (95% confidence interval (CI): 0.9-1.7) (31). The results of these studies were similar to our findings.

Self-rated health status was significantly negatively associated with a depressive tendency in diabetic women in our study. A Canadian study of 1265 patients with type 2 DM discovered an adjusted 2.05-fold increase in the risk of depression in people with poor self-rated health (95% CI: 1.20-3.48) (32), and studies in the United States and Germany have identified a similar result (27,33,34).

Social and economic situations should be related to depressive tendency. Studies have shown that individuals with lower socioeconomic status, unskilled workers, and retired people have a higher depressive tendency (25,26). Another study found a higher risk of depression in unemployed people (odds ratio: 1.99, 95% CI: 1.04-3.81) and the poor (odds ratio: 1.52, 95% CI: 1.01-2.28) (35). However, a small-sample-size study reported that social and economic situations are not related to depressive tendency (36). The results of our study were that employment status was related to the tendency toward depression and that unemployed people had higher CES-D scores.

The relationship between degree of education and depressive tendency remains controversial. Some studies have suggested that individuals with lower education levels have a higher depressive tendency (25,34,37), whereas one study did not find this relationship (36). Our results demonstrate that a higher education level (college or above) was related to a higher depressive tendency.

Different studies have obtained different results regarding the relationship between marital status and depressive tendency. Studies showed that widowed persons (28), unmarried people (38), divorced (28,39), and separated individuals (39) had a higher tendency toward depression. Our study found no evidence of this association, which was consistent with the result of two studies (34,36). The relationship between age and depression in patients with diabetes is debatable. Although some studies have shown that the age was not related to depression (40,41), others have shown that older diabetic patients had a higher depressive tendency (26,42), and yet others have found that younger diabetic patients have a higher tendency (27,34,35,37). We did not find a significant relationship between age and depression in the present study.

The relationship between duration of DM and depressive tendency has also been inconsistent. One study demonstrated that having DM for more than 5 years was related to depression (37). Another found DM for more than a decade was related to depression (adjusted odds ratio: 3.70, 95% CI: 1.09-14.29) (43). There are contrasting results on whether the duration of DM is significantly associated was (44) or was not associated with (40) depressive tendency. Depressive tendency was found to be independent of insulin usage (34,41); however, one study stated that it was related to the pattern of treatment in diabetic patients (39). Our results demonstrated that patterns of treatment and duration of DM were not related to depressive tendency. Depressed individuals appear to have worse glucose control (28,38,40,44,45). Our results, however, demonstrate that glucose control was not related to depressive tendency, which was consistent with the result of one previous study (46).

### Study limitations

Due to the cross-sectional nature of this study, there may have been selection bias, and causal relationships could not be investigated; our study could only explore the correlation between HL and depressive tendency in diabetic women. Under limited resources, we could only recruit 152 women, and our results may have been limited by our small sample size. Most of our participants were mid-aged and younger than another study in Chiayi, Taiwan (28). Only 14.07% were more than 65 years old in our study compared to 68.7% in the previous study (28). Our sample may not represent most of the diabetic women in Taiwan. However, our results did not change after adjustment and stratification based on age. Moreover, there may be educational differences in the validity of a self-reported questionnaire (47) and we did not approach this problem. Application of our results should be restricted to women with type 2 DM with backgrounds similar to those of the women in our study due to its cross-sectional nature.

## CONCLUSION

HL was significantly associated with a depressive tendency among diabetic women in our study. Enhancing HL of diabetic women may reduce the psychological distress of those who suffer from depression. Further interventional studies are needed to establish the effect of improved HL on depression. Developing skills to improve their HL should be the focus of further strategies.

## AUTHOR CONTRIBUTIONS

Hsu YL conceived and designed the study, was responsible for acquisition, analysis, and interpretation of data, drafted the manuscript and approved the final manuscript version to be published. Su DH participated in the analysis and interpretation of data, critically revised the manuscript for important intellectual content and approved the final manuscript version to be published. Kuo SC participated in the design of the study and critically revised the manuscript for important intellectual content and approved the final manuscript version to be published.

## Figures and Tables

**Table 1:** Demographic characteristics of the patients.

Factors	Number/percentage or Mean ± standard deviation	Group CES-D scores	Test value	*p*
Age	54.66±11.43 (22∼87)		-0.017^1^	0.84
Education level			1.182^2^	0.76
Elementary	29 (21.48%)	12.10±8.98		
Junior high	28 (20.74%)	10.86±8.67		
Senior high	44 (32.59%)	9.23±7.30		
College or above	34 (25.19%)	11.94±9.89		
Marital status			2.501^2^	0.11
Unmarried + Widowed	14 (10.36%)+6 (4.45%)	14.15±10.13		
Married + Divorced	109 (80.74%)+6 (4.45%)	10.30±7.97		
Religious belief			-0.12^3^	0.91
Yes	98 (72.59%)	10.92±8.78		
No	37 (27.41%)	10.73±8.26		
Employment status			3.464^2^	0.063
Unemployed	22 (16.30%)	13.95±9.64		
Employed + Housewife	66 (48.89%)+47 (34.81%)	10.27±8.77		
Monthly income			0.653^2^	0.72
Below USD 1208	40 (29.63%)	9.83±7.22		
Above USD 1208	18 (13.33%)	11.33±7.59		
No	77 (57.04%)	11.30±9.17		
Economic satisfaction			7.533^2^	0.007*
Living beyond one’s income + Living well within income	13 (9.63%)+2 (1.48%)	18.13±12.01		
Living at income level + Living within income	86 (63.70%)+34 (25.19%)	9.96±7.58		
Dependence status			-1.00^3^	0.317
Dependent on others	34 (25.19%)	12.11±9.22		
Self-care	101 (74.81%)	10.45±8,42		
Companion			0.016^2^	0.90
Family	127 (94.07%)	10.80±8.32		
Others	8 (5.93%)	12.00±10.17		

1 Pearson’s correlation coefficient

2 Kruskal–Wallis test

3 t-test

* statistically significant; USD: United States Dollar.

CES-D: Center for Epidemiologic Studies Depression Scale.

**Table 2:** Clinical characteristics of the patients.

Factors	Number/percentage or Mean ± standard deviation	Group CES-D scores	Test value	*p*
The duration of diabetes	1∼32 years 7.27±6.12 years		-0.004^1^	0.98
Longer duration of diabetes			0.26^3^	0.79
<=10 years	104 (77.04%)	10.97±9.35		
>10 years	31 (22.96%)	10.52±7.29		
Insulin using			0.11^3^	0.91
No	102 (75.56%)	10.91±9.23		
Yes	33 (24.44%)	10.73±7.89		
HbA1C	5.2∼14.7% 7.91±1.69%		0.016^1^	0.86
Good sugar control			-0.28^3^	0.78
HbA1C<=7%	42 (31.11%)	11.17±8.85		
HbA1C>7%	93 (68.89%)	10.73±8.23		
CES-D	0∼36 points 10.87±8.40 points			
Depression tendency				
Low (CES-D<16)	107 (79.26%)			
High (CES-D≧16)	28 (20.74%)			
CHLSD	14-68 points 59.70±8.55 points		-0.181^1^	0.036*
Health literacy			2.584^2^	0.108
Insufficient (CHLSD≦53)	18 (13.33%)	14.72±10.46		
Enough (CHLSD>53)	117 (86.67%)	10.27±7.92		
Self-rated health status	0∼10 points 5.89±1.70 points		-0.280^1^	0.001*

1 Pearson’s correlation coefficient

2 Kruskal–Wallis test

3 t-test

* statistically significant.

CES-D: Center for Epidemiologic Studies Depression Scale; CHLSD: Chinese Health Literacy Scale for Diabetes.

**Table 3:** Linear regression model of depressive tendency and health literacy of women with diabetes.

Factors	Univariate analysis			Multivariate analysis		
	Parameter	Test value	*p*	Parameter	Test value	*p*
Age	-0.013	-0.20	0.84			
Education level (base: primary + high school)						
College or above	1.44	0.86	0.39	2.88	1.83	0.069
Marital status (base: married + divorced)						
Unmarried + widowed	3.85	1.91	0.06			
Religious belief (Base: no)						
Yes	0.19	0.12	0.91			
Employment level (base: unemployed)						
Employed + housewife	-3.69	-1.90	0.059	-3.74	-2.03	0.044*
Monthly income (base: No)						
Below USD 1208	-1.47	-0.90	0.37			
Above USD 1208	0.035	0.02	0.99			
Economic satisfaction (base: living at income level + living within income)						
Living beyond one’s income +living well within income	8.18	3.72	0.003*	8.27	3.92	0.0001*
Dependence status (base: self-care)						
Dependent on others	1.67	1.00	0.32			
Companion (base: family)						
Others	1.20	0.39	0.70			
Longer duration of diabetes (base:<=10 years)						
>10 years	-0.46	-0.26	0.79			
Insulin using (base: no)						
Yes	-0.18	-0.11	0.91			
Good sugar control (base: HbA1C>7%)						
HbA1C<=7%	0.44	0.28	0.78			
Health literacy	-0.18	-2.12	0.04*	-0.17	-2.05	0.042*
Self-rated health status	-1.39	-3.37	0.001*	-1.11	-2.85	0.005*

* significant.

USD: United States Dollar.
